# Supplementary material for: Enhanced Photovoltaic Performance of Inverted Perovskite Solar Cells through Surface Modification of a NiOx-Based Hole-Transporting Layer with Quaternary Ammonium Halide–Containing Cellulose Derivatives
Source: Polymers (Basel). 2023 Jan 13;15(2):437. doi: 10.3390/polym15020437 (PMC9862003; doi:10.3390/polym15020437)
Supplement: Supplementary file 1 [file polymers-15-00437-s001.zip › polymers-2107404-supplementary.pdf]

Enhanced photovoltaic performance of inverted perovskite solar cells through surface modification of a NiO_x-based hole-transporting layer with quaternary ammonium halide–containing cellulose derivatives

I-Hsiu Ho[1], Yi-Jou Huang[1], Cheng-En Cai[1], Bo-Tau Liu[2], Tzong-Ming Wu[3], and Rong-Ho Lee[1]*

1. Department of Chemical Engineering, National Chung Hsing University, Taichung 402, Taiwan, Republic of China
2. Department of Chemical and Materials Engineering, National Yunlin University of Science and Technology, Yunlin 64002, Taiwan, Republic of China
3. Department of Materials Science and Engineering, National Chung Hsing University, Taichung 402, Taiwan; tmwu@nchu.edu.tw

*To whom correspondence should be addressed

Rong-Ho Lee—e-mail: rhl@dragon.nchu.edu.tw; tel.: +886-4-22854308; fax: +886-4-22854734

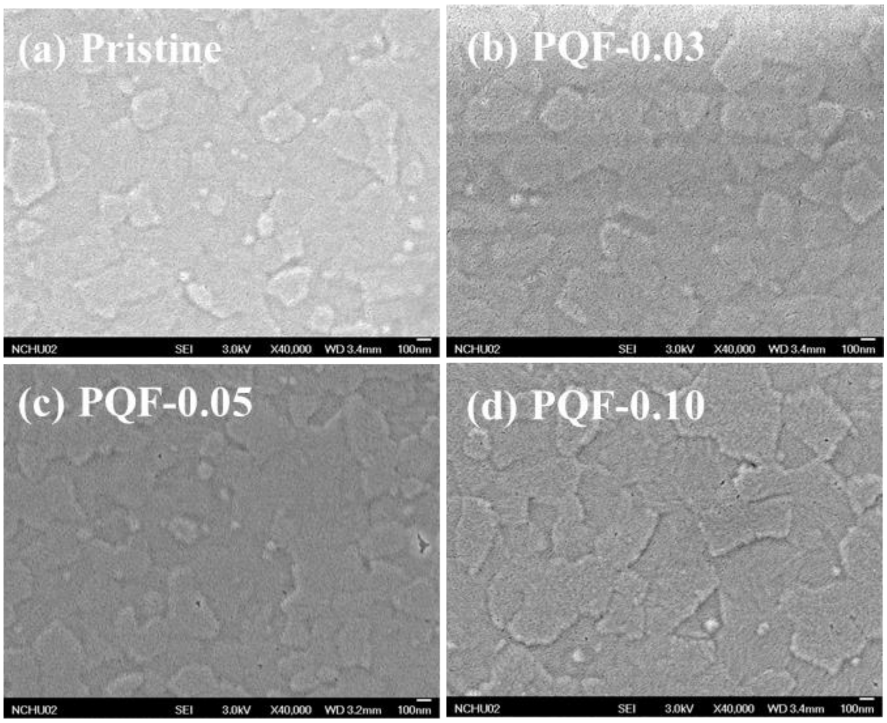

Figure S1. SEM images of the PQF-deposited NiO_x layers: (a) pristine, (b) PQF-0.03, (c) PQF-0.05, and (d) PQF-0.10.

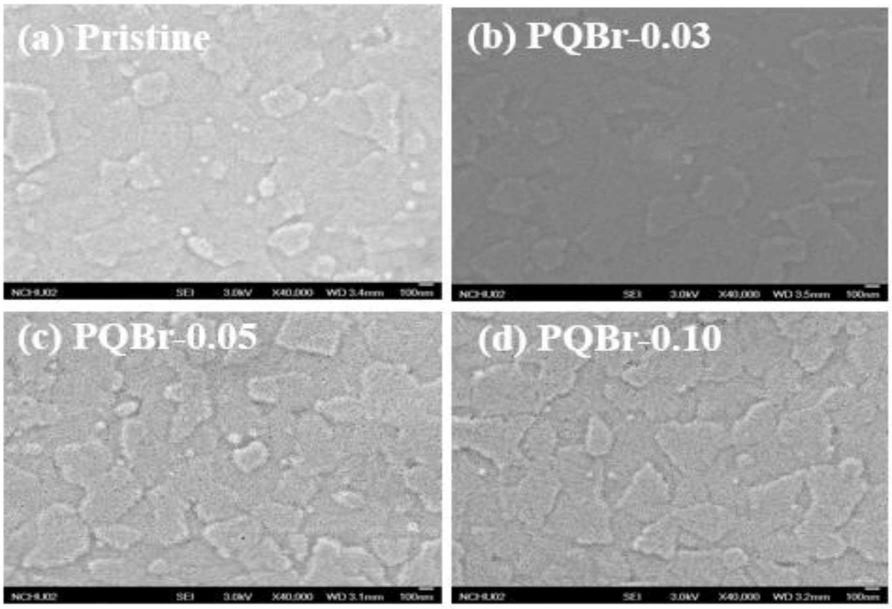

Figure S2. SEM images of the PQBr-deposited NiO_x layers: (a) pristine, (b) PQBr-0.03, (c) PQBr-0.05, and (d) PQBr-0.10.

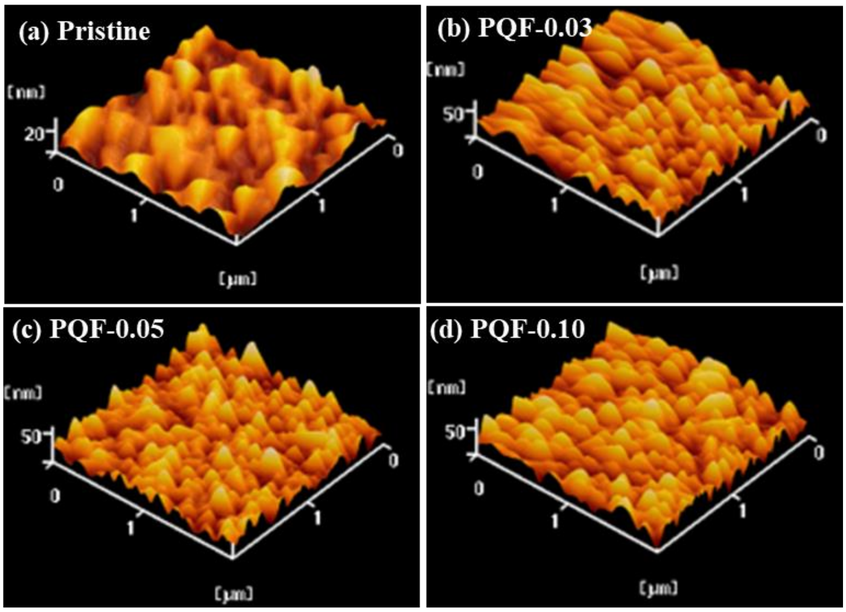

Figure S3. Topographic images of PQF-deposited NiO_x layers: (a) pristine, (b) PQF-0.03, (c) PQF-0.05, and (d) PQF-0.10.

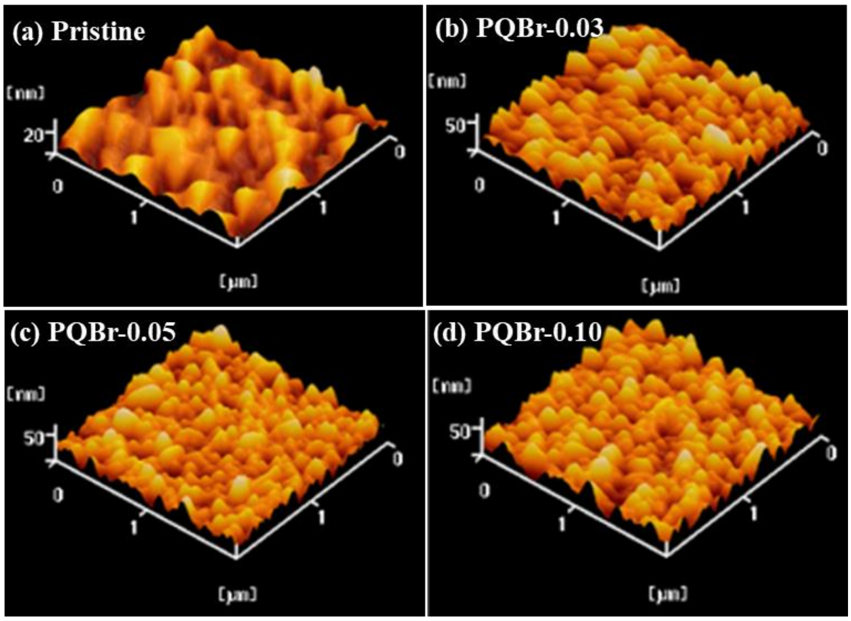

Figure S4. Topographic images of PQBr-deposited NiO_x layers: (a) pristine, (b) PQBr-0.03, (c) PQBr-0.05, and (d) PQBr-0.10.

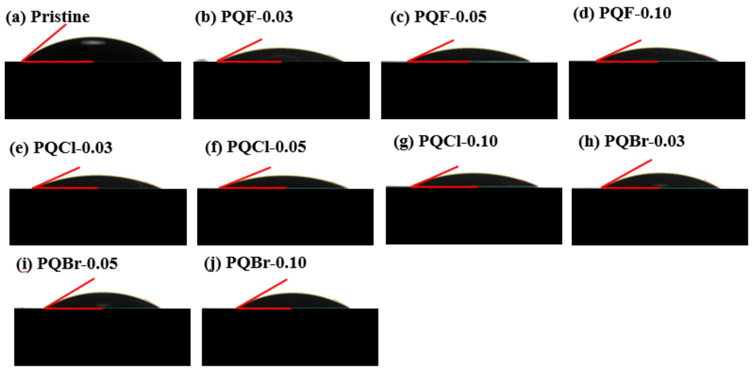

Figures S5. Photographs of water droplets on the pristine and cellulose derivative–deposited NiO_x layers.

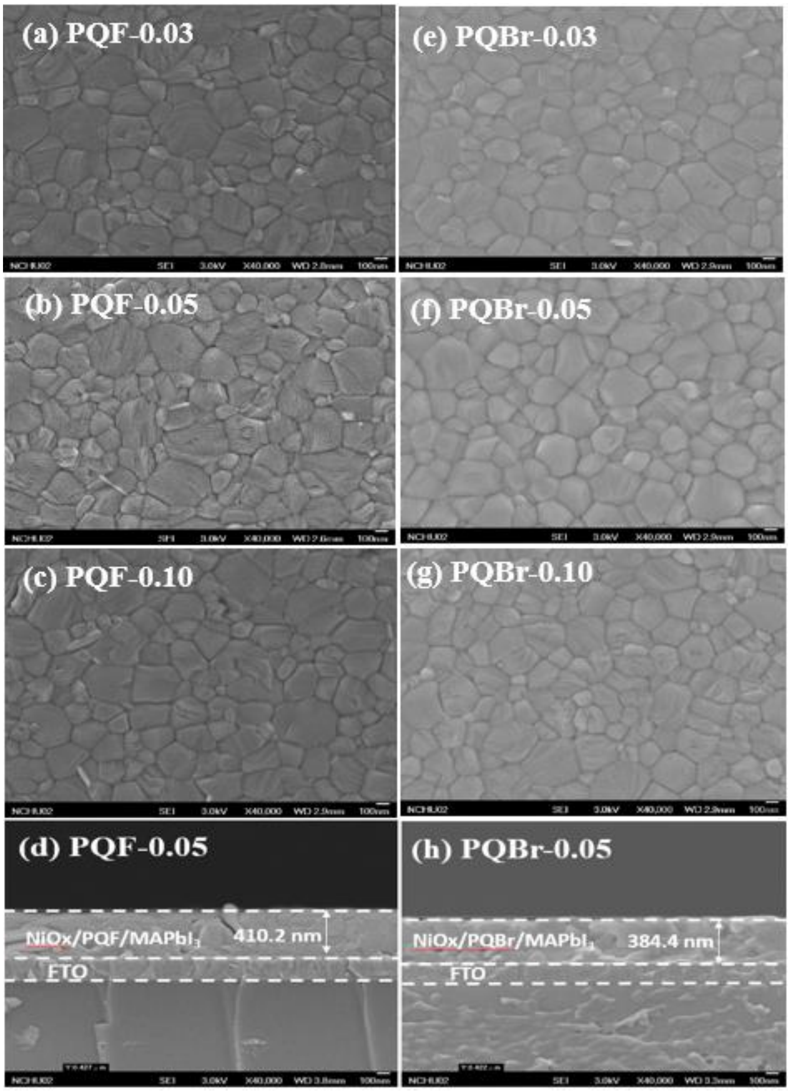

Figure S6. (a–c, e–g) Top-view and (d, h) cross-sectional SEM images of (a) PQF-0.03–, (b, d) PQF-0.05–, (c), PQF-0.10–, (e) PQBr-0.03–, (f, h) PQBr-0.05–, and (g) PQF-0.10–based perovskite films.

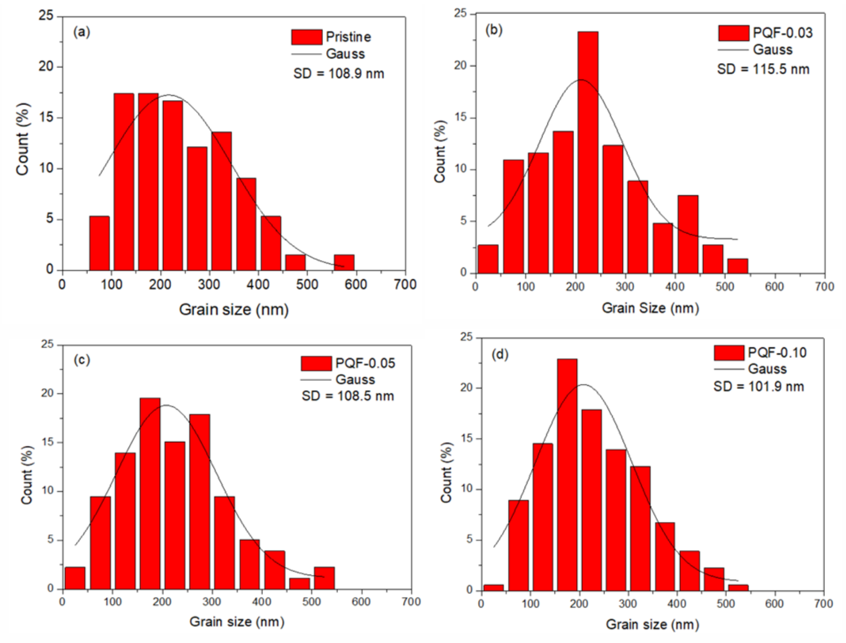

Figure S7. Grain size statistics and Gauss fits of the crystal grain size distributions of pristine and PQF-0.03–, PQF-0.05–, and PQF-0.10–based perovskite films (SD: standard derivation).

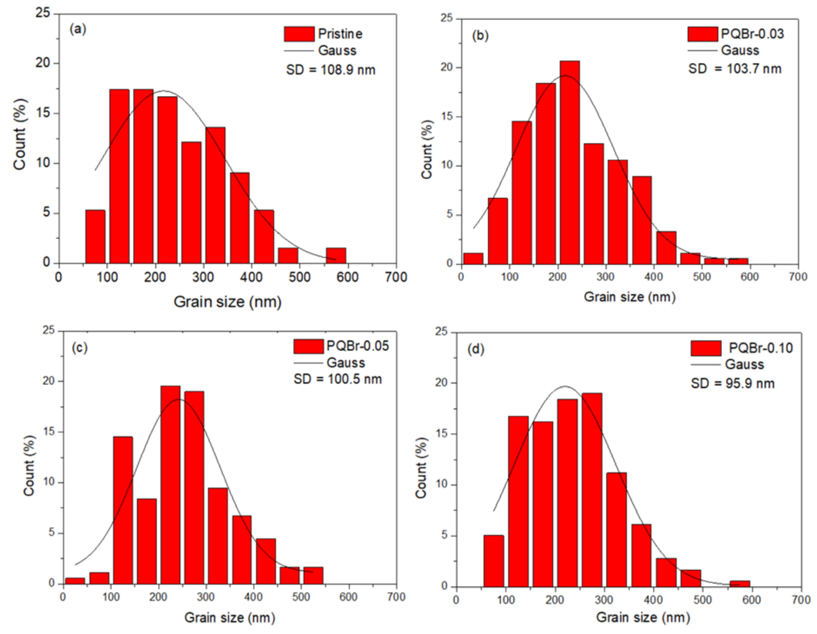

Figure S8. Grain size statistics and Gauss fits of the crystal grain size distributions of pristine and PQBr-0.03–, PQBr-0.05–, and PQBr-0.10–based perovskite films (SD: standard derivation).

.

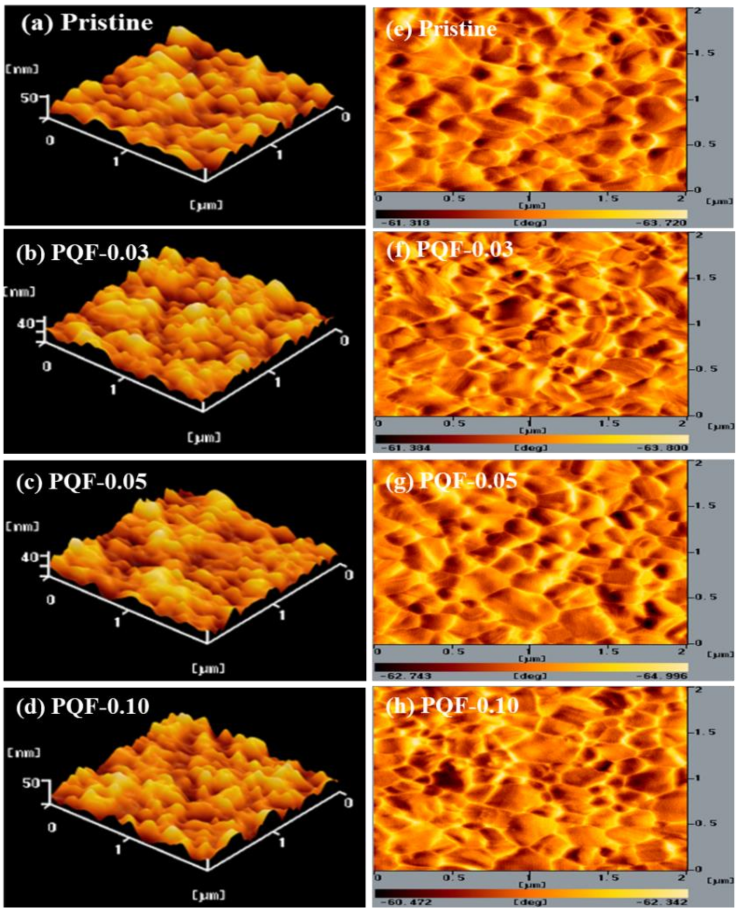

Figure S9. (a–d) Topographic and (e–h) phase AFM images of perovskite films deposited on (a, e) pristine and (b, f) PQF-0.03–, (c, g) PQF-0.05–, and (d, h) PQF-0.120–modified NiO_x.

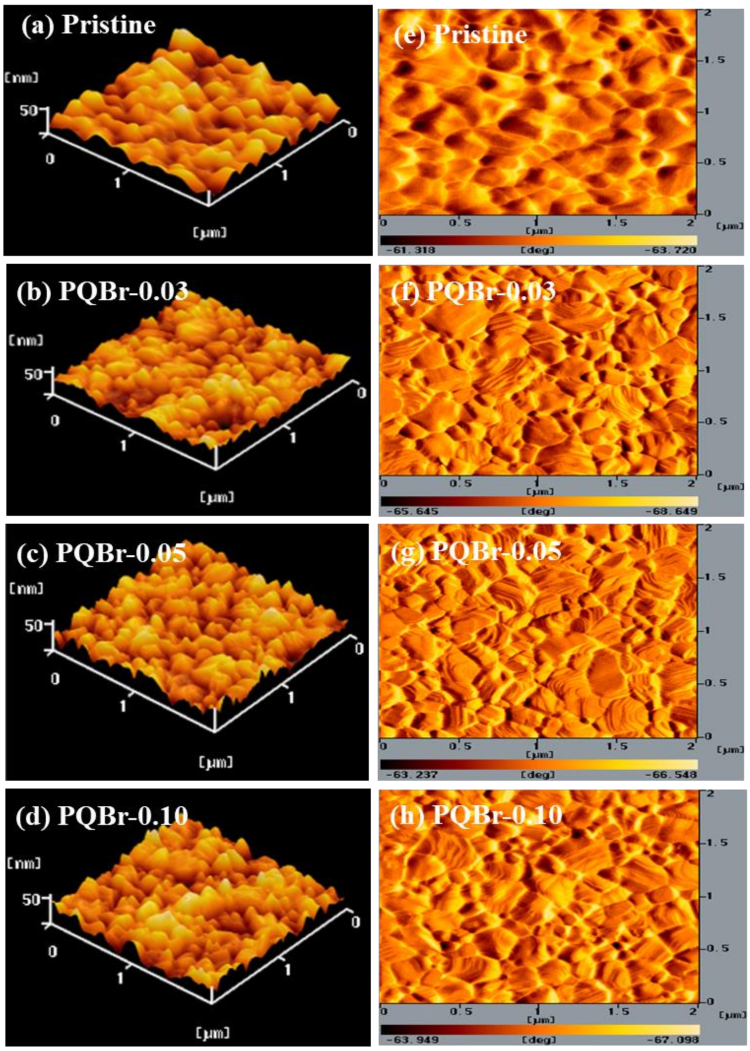

Figure S10. (a–d) Topographic and (e–h) phase AFM images of perovskite films deposited on (a, e) pristine and (b, f) PQBr-0.03–, (c, g) PQBr-0.05–, and (d, h) PQBr-0.10–modified NiO_x.

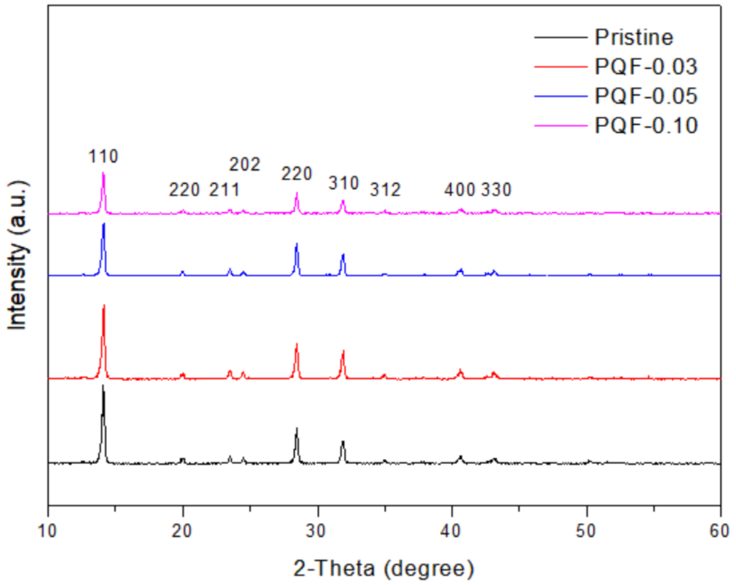

Figure S11. XRD patterns of $MAPbI_3$ films deposited on PQF-modified NiO_x layers.

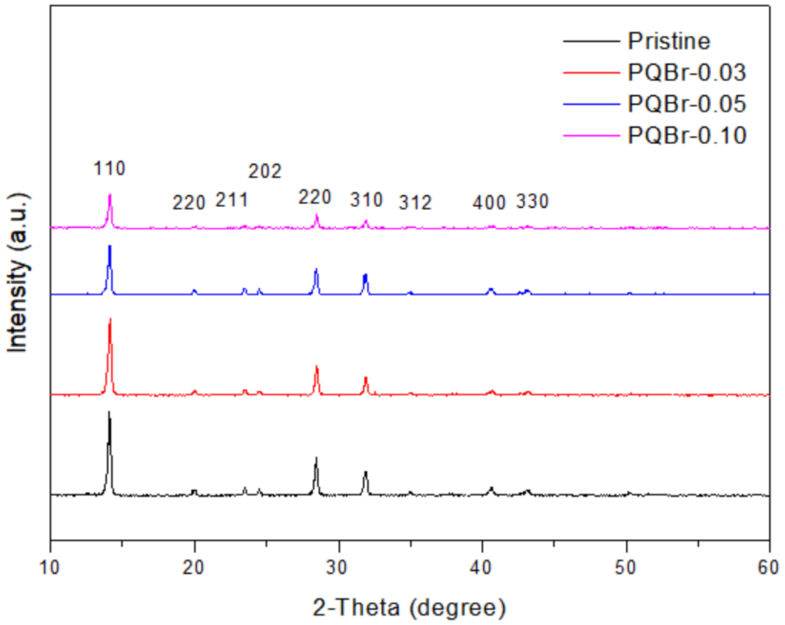

Figure S12. XRD patterns of $MAPbI_3$ films deposited on PQBr-modified NiO_x layers.

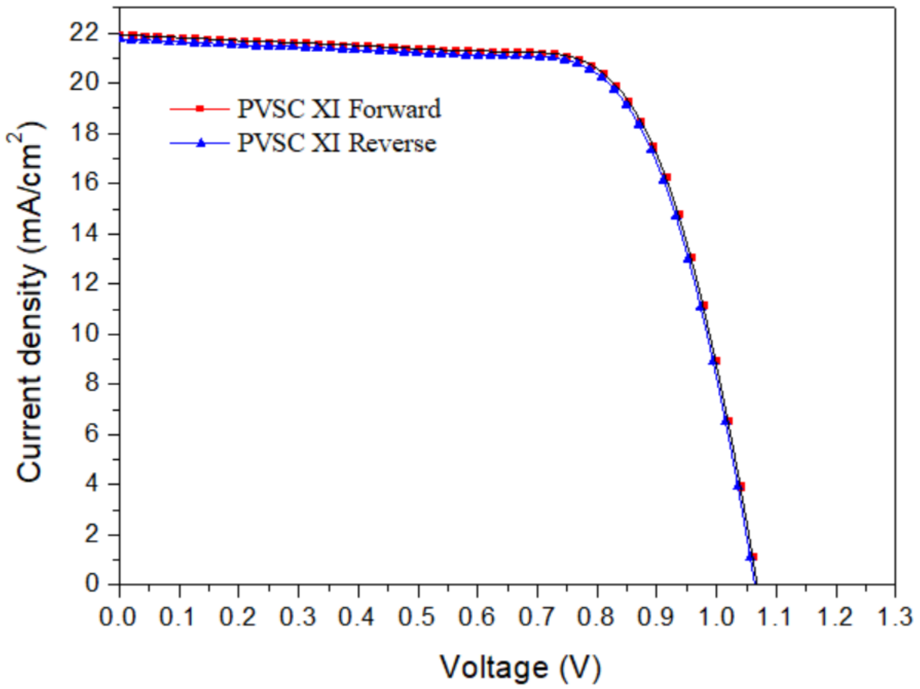

Figure S13. Current density–voltage curve hysteresis of the PVSC XI.
